# Dental Diseases and Our Responsibilities

**Published:** 1913-06-15

**Authors:** N. S. Hoff


### DENTAL DISEASES AND OUR RESPONSIBILITIES.

BY N. S. HOFF, D. D. S.

Read before the Northern Michigan Dental Society June 7th, 1913.

The statement frequently made, that dental caries is the most prevalent disease known to mankind, can be conclusively verified by modern statistics. That it exists in every civilized nation of the world is positively known, and that it prevails to an almost incredible extent in the most civilized and cultured countries and communities can also be prooved. That it is more prevalent in some classes of people than in others in the same country has also been demonstrated. Reliable statistics gathered in communities of average social standing in Germany, France, and England, by scientific examinations of thousands of children have shown a general average of dental caries of 95% of all the children examined. The same kind of statistics in the United States gives a slightly better condition of oral health, namely 92%. In the most highly cultured communities of this and other countries, this average has been reduced only to about 85%.

Dental caries has sometimes been said to be a children's disease; and while this statement is very largely true, we find it also very prevalent in the teeth of our adult and older patients. In the older persons, it is not excessive, and usually appears periodically, or is contrasted with periods of practical immunity varying from complete cessation to conditions of more or less freedom from threatening prevalence. Caries may be absent entirely for years and then recur or it may recur at more frequent intervals. Just why caries is more prevalent in children's teeth, and why it should prevail at one time in the adult mouth and then disappear for months or years to again recur in the same mouth, are the problems that have for many years engaged our keenest thoughts and tempted our scientists to the most painstaking researches to discover, with as yet no satisfactory explanation. We know something of how the process is accomplished and we have learned by persistent effort to combat and overcome its ravages, but we are still seeking the secret of the why, which will give us the clue as to its prevention.

Scribonius Largus, a learned Roman physician about 50 A. D. wrote that decay of the teeth was produced by worms working in the teeth. He had no idea of the modern microbic theory, but probably took his cue from the ancient Chinese idea that worms caused decay of the teeth. This worm theory, for lack of a better one, appeared in ancient literature more or less persistently for sixteen centuries.

Claudius Galen about 175 A. D. wrote that dental caries was due to acrid humors in the tooth itself. This theory was sanctioned as late as 1728 by the great Fauchard, who could not believe the worm theory, and could in no other way account for it.

Dr. George Watt, of Ohio, about 1860 promulgated what was known as the "mineral acid theory." He made such a plausible explanation of his theory, that it became the accepted theory for the next twenty-five years. Substantially his theory was, that the acids were formed in the mouth from foods collecting in the fissures and inter-dental spaces where by chemical disintegration the acid was set free from its compounds in a nascent form, in contact with the tooth tissue, and being protected by the masses of foods and extraneous adhesions, it was not easily diluted by the saliva, and so it attacked the calcic tissues and etched its way into the tooth. To explain the various forms and colors of carious teeth he claimed the black decay was caused by sulphuric acid which was formed in the mouth by the disintegration of foods containing sulphur, eggs, etc. The brown decay was produced by hydrochloric acid, formed in a similar way, by decomposition of sodium chloride, and regurgitation of stomach juice, etc. The white decay was produced by nitric acid, formed from the union of ammonium and oxygen in the mouth. He also claimed that erosion was a chemical process caused by the fruit acids. This theory was certainly ingenious and might well have continued to satisfy, had not Koch discovered the function of micro-organisms as causative factors of disease, and had not Prof. W. D. Miller made his great research which conclusively proved the manner in which dental caries was produced by fermentation, and how the destructive acids were really formed This made the whole subject of the etiology of dental caries perfectly clear, and established its etiology for all time. However, Miller did not get so far in his work as to determine why caries prevails at the several periods mentioned above, and why it did not occur at other periods in life's history. Neither did he, nor could he, give us a method of positive prevention. As we have said, our scientists are still at work on this problem. Possibly if our beloved Miller had lived, he would have completed his work and given us this much to be desired clue to the complete eradication of this scourge of human kind.

In the mean time we are doing what we can to repair the damages as they accumulate, and by constant vigilance, we believe we are in a commendable measure stemming the tide of this disease by our technical endeavors, and by such sanitary suggestions as we are enabled to enforce with the few patients whom we are permitted to serve and influence. But when we realize that by the usual repair methods the average dentist can not hope to serve properly, that is, with necessary treatment, more than 200 patients in a year, which would mean one day and a half to each, the subject looms up in terrifying proportions. Because of the limited number of practitioners, every dental practitioner should serve 2400 people each year, that is, he should render all the dental services 8 people will need for an entire year in one day. Is there any dentist who believes such a task is possible? Besides, conditions are growing worse every day. The number of dentists is not increasing in relative proportions to the increase in the population, intelligence and demand for our services. Tooth manufacturers tell me that more artificial teeth are being manufactured today than ever before in the history of the world. Most dentists are confining their efforts to saving the teeth of a few favored people, but the masses are loosing their natural teeth and the advertising dentists and commercial laboratories are doing their best to take care of the results of the most prevalent disease known to our civilization.

The other disease whose treatment comes wholly within the scope of the dentist, while not so universally prevalent as dental caries, and which instead of being classed as a children's disease, may well be called the old man's disease, because few old people escape it. This disease has not as yet been scientifically studied and classified. In fact, we do not yet know it well enough to intelligently name it. We have named it for men who have tried to treat it, and we have tried to name it according to its symptomatic manifestations, and from our various hypothetical conseptions of its etiology. We have called it "Rigg's Disease," because Dr. Riggs first attempted and claimed he could cure it, and devised a most successful surgical treatment for it, about 1875. It is an ancient trouble, like dental caries. It is neither contagious, nor infectious, and consequently never epidemic. It is however universally prevalent and as old as history. The ancient Egyptians and Phoenicians suffered from it and evidence of ingenious efforts to repair its ravages supply or most important evidences of ancient prosthetic dentistry in the form of bridge work. Pliny the Second, a great Roman scholar, about 70 A. D. wrote extensively of loose teeth and collected much information as to methods of treatment. It seems to have been most prevalent among tha class living idle and luxuriant lives, particularly the Romans about the time of Nero, and later.

Among more modern writers, F. Ruysch, a Dutchman in 1725 described loose teeth and attempted treatment. He called the disease Scurvy, and said it was caused by excessive diet, or faulty diet, and from deposits of salivary tarter on the roots of the teeth. Calvi, a Frenchman, in 1860 described it with much accuracy, and named it "Pyorrhea inter alveol dentaire." Since then it has received much consideration, and has been called "Infectious Alveolitis" by Witzel, who wrote of it in 1882. Black in 1886 wrote a work on the peridental membrane, and called this trouble "Phagedenic Pericementitis." Since then many authors have attempted to classify and name it, but with more or less unsatisfactory results. The disease is not clearly made out and expresses itself in so many different forms that it can probably be no better known than by the name of Peridentitis, which is capable of expressing the various symptoms by a qualifying adjective, such as Gingival Peridentitis, Carious Peridentitis, Gangrenous Peridentitis, Suppurative Peridentitis, etc. The usual name, Pyorrhea Alveolaris, is not sufficient as it expresses but one stage or symptom of the disease. But whatever we call it, the disease is with us and we must discover its cause and undertake its treatment and provide our patients and the public with such information as shall enable them to apply preventive measures when practicable. It is much to the discredit of our profession that so many consider this disease incapable of successful relief by either our surgical or therapeutic resources. While we do not possess such definite scientific knowledge as to the etiology of this disease, we have accumulated considerable clinical data as to its treatment. It seems to be the general attitude of the profession that this disease is incurable when thoroughly established in the mouth. There are however, a large number of practitioners who by patient endeavors have satisfied themselves that it is a curable disease by treatment that is within the possible range of practitioners of good surgical and therapeutical attainments. We hav'nt space or time here to discuss the numerous forms of treatment advocated, but there can be no doubt that much can be done to stay the ravages of this disease, and when the tissues are somewhat desparately involved, rescue and restoration treatment can be definitely secured in so large a majority of cases, that enthusiastic workers with this disease claim that few cases are absolutely hopeless. In fact, so much in the way of rescue treatment has been successful that the prevention of the disease seems most probable.

Another disease which we cannot discuss in detail, but which has a somewhat direct bearing on the purpose of this paper should be mentioned at least, and that is the disease embracing numerous complications which has been somewhat widely discussed lately under the name of "Oral Sepsis." The recent agitation of the profession because a physician saw fit to attribute many severe nervous disorders to faulty dentistry, called our attention so sharply to the fact that severe systemic disorders are not only frequently associated with dental diseases, but also to the more important fact that dental diseases may be the direct cause of serious systemic diseases. We have long known that eye, ear, nose and throat troubles may be caused or at least aggrevated by dental diseases. We are now learning that insomnia, insanity, and mental incapacity may arise from dental irritation. We have long recognized the fact that neuralgia, headache, and tic douloureux are due to irritation of the dental branches of the trigeminus nerve. We have also recognized the reactions on the whole alimentary tract of pain in the teeth. A more insidious form of systemic disorder seems now to demand our attention, because of the influence on general body health of certain forms of dental treatment, especially the adaptation of crowns and bridges to vital and devitalized teeth. Because of imperfect manipulation or the neglect to operate under strict aseptic conditions, foci of infection are provided at the apical part of the tooth, in an environment which does not permit of rapid or noticable local irritation, but nevertheless it steadily persists and while not noticed by the patient, it expresses itself by referring the pain to some other region and afflicts an organ or tissue so severely and persistently that its cause and diagnosis becomes exceedingly difficult and the treatment is baffling to the physician. Such conditions are not always caused by imperfect dental operations, but may be the outcome of diseased teeth, or roots, which, from exposed and infected pulps, or from dormant abscesses, or necrosis, become the active cause of functional disorder in some remote organ or tissue, which may be in a condition of low physicial resistance, predisposing it to such harmful influences. When we stop to think of the innumerable sources of infection from a mouth filled with diseased teeth we wonder how any person of mature existance can be healthy because of the universal presence of alveolar diseases and so much bad dentistry.

There are other complications and accidental interferences that would help to make a statement of larger proportions if it were needed to show the importance of the oral cavity in any well considered plan or system of general or personal hygiene, but the three important factors which we have presented ought to be sufficient to convince the most skeptical that we as a profession, must take a broader outlook of our duty, and exercise more vigilance in preventing all such unwholesome conditions, not only in the mouths of our own patients, but we must call the physician's attention to any dental or oral affection which may have a bad systemic influence, in order that he may make his diagnosis more complete and become more intelligent and successful in his treatment. The great campaign of public school and institutional inspection of children's teeth, and the wholesale efforts to instruct these children as to correct methods of oral sanitation is doing a powerful lot of good and its influence will go on for many years to come and probably result in better teeth in future generations. We all rejoice that it is going on so grandly and that members of our profession are willing to give of their time as well as their money to help on this grand cause. The universal prevalence of dental caries, peridentitis and oral sepsis will undoubtedly receive a much to be desired check because of this movement but will preventive dentistry ever be realized in a large measure by any such public propaganda? We fear not! The religious revival serves to interest people in their soul's welfare, the political campaign is essential to the development of sentiment for or against a man, or the party he represents; and so in other well known spheres of activity, the public as such, must first be awakened to its needs by the singing of hymns and the torch light processions, but it is the hand to hand methods that count for results after all, and, so I believe in our present campaign for preventive dentistry, the next step that must be taken should be by the Dental Profession; not as an army fighting en masse and in the open field, but each man in his own province, and by the most tactful and effective methods he can command. Every patient should be surcharged with information as to the value of his teeth, and be made fully acquainted with the conditions prevalent in his own mouth which are possible if not probable factors of ill health. He should be made aware of the necessity for the most careful and complete treatment available, and be shown the reasons for this because of his own and the worlds best progress to the ideal condition of health and general efficiency.

As practitioners we have cause to know the injury that usually comes to our patients from the loss of even a single tooth, we never want our patient to loose the first tooth, but when several teeth are lost we get used to it and seem to think one tooth more or less does not make much difference. We have not yet come to understand the inestimable value of a perfect set of teeth and the influence such denture may have in conserving the normal health and functions of the entire body. When we do realize this fact and conscientiously study our relations as conservors of health in our daily practice of the profession, we shall then assume a more responsible and aggressive attitude towards these diseases, and naturally we shall serve our calling in a more professional manner. Then we shall begin our ministration with the child as soon as he has teeth, or perhaps before, and we shall insist that our advice and instructions as to personal care of the teeth are systematically and efficiently carried out. To do this we have a large campaign of education before us. We must take the parents of children into our confidence and have their cordial cooperation. We must fill carious cavities in decidious teeth; see that the teeth are extracted at the proper time only; enforce oral prophylaxis at the earliest possible date; prevent mal-occlusion; keep the teeth of the permanent set under constant surveilance from the eruption of the first molar to the final appearance of the third molar. If by such means we can carry the youth to maturity without loss of any tooth, and with his teeth in normal alignment, we shall save the day for such a patient and have taught him habits of personal hygiene that will serve to keep him free from dental loss or general systemic complications, and we shall make him a healthy and normal citizen, instead of permitting him to become a physical and mental bankrupt.

If now this can be done, and every one who has studied this question at all closely believes it practicably possible, is'nt it time for us to take a new view of our duty and make such changes in our attitudes to oral hygiene as to accomplish this ideal conception of our duty and privilege as a profession which has as noble opportunities for ministration as any of the older professions? The opportunity is at hand; the need and the means are known to us; our patients are beginning to inquire what we are going to do about prevention. Shall we shirk a plain duty and loose our inheritance?

